# Development and In Vitro Evaluation of Lyotropic Liquid Crystals for the Controlled Release of Dexamethasone

**DOI:** 10.3390/polym9080330

**Published:** 2017-08-02

**Authors:** Márcia H. Oyafuso, Flávia C. Carvalho, Tatiane M. Takeshita, Ana L. Ribeiro de Souza, Daniele R. Araújo, Virginia Merino, Maria Palmira D. Gremião, Marlus Chorilli

**Affiliations:** 1 School of Pharmaceutical Sciences, São Paulo State University (UNESP), 14800-903 Araraquara, Brazil; marcia.oyafuso@yahoo.com.br (M.H.O.); tati.mtakeshita@gmail.com (T.M.T.); analuizars@yahoo.com.br (A.L.R.d.S.); 2 School of Pharmaceutical Sciences, Federal University of Alfenas, UNIFAL-MG, 37130-000 Alfenas, Brazil; flaviachiva@gmail.com; 3 Human and Natural Sciences Center, Federal University of ABC, 09210-580 Santo André, Brazil; daniele.araujo@ufabc.edu.br; 4 Instituto Interuniversitario Reconocimiento Molecular y Desarrollo Tecnológico, Departamento de Farmacia y Tecnología Farmacéutica y Parasitología, Universidad de Valencia, 46010 Valencia, Spain; Virginia.merino@uv.es

**Keywords:** amphiphilic polymers, lyotropic liquid crystals, controlled release, drug release, kinetic model, dexamethasone, nanostructured systems

## Abstract

In this study, amphiphilic polymers were investigated as biomaterials that can control dexamethasone (DXM) release. Such materials present interfacial properties in the presence of water and an oily phase that can result in lyotropic liquid crystalline systems (LLCS). In addition, they can form colloidal nanostructures similar to those in living organisms, such as bilayers and hexagonal and cubic phases, which can be exploited to solubilize lipophilic drugs to sustain their release and enhance bioavailability. It was possible to obtain lamellar and hexagonal phases when combining polyoxyethylene (20) cetyl ether (CETETH-20) polymer with oleic acid (OA), *N*-methylpyrrolidone (P), isopropyl myristate (IM), and water. The phases were characterized by polarized light microscopy (PLM), small-angle X-ray scattering (SAXS), rheological, textural, and bioadhesion analyses followed by an in vitro release assay. All samples showed elastic behavior in the rheology studies and hexagonal samples containing P and IM showed the highest adhesiveness. The drug release profile of all LLCS presented an average lag time of 3 h and was best fitted to the Korsmeyer-Peppas and Weibull models, with controlled release governed by a combination of diffusion and erosion mechanisms. These systems are potential carriers for DXM and can be explored in several routes of administration, providing potential advantages over conventional pharmaceutical forms.

## 1. Introduction

Dexamethasone (DXM) is a first-line glucocorticoid drug with a mechanism of action related to the regulation of the expression of inflammatory cytokines (IL-1b, IL-6, IL-10, IL-1RA, TNF-a, and IFN-g), chemokines (CXCL-10 and CCL-5), lipocortin, metalloproteinases (MMP-1, MMP-2, MMP-3, MMP-9, and MMP-13), and TIMP-1 [1]. Oral or intravenous administration presents limitations, such as low bioavailability that demands high doses, resulting in serious systemic side effects. Therefore, DXM is most often used for ocular or inflammatory skin diseases, although it exhibits poor permeability through the corneal epithelium and stratum corneum [1,2]. However, those limitations can be overcome through various drug formulation strategies. New topical formulations are also necessary for wound healing, and DXM has been tested as a drug model [3,4,5]. In addition, DXM has been used in scaffolds with controlled pore structures for osteogenic differentiation of mesenchymal stem cells; research on new drug delivery systems for the controlled release of DXM is necessary [6]. Nanotechnology and bioadhesive polymers have great potential for prolonged control and targeted corticosteroid delivery, making them an appropriate vehicle to improve treatment efficacy [2].

Liquid-crystalline systems are potential vehicles for the topical administration of drugs with solubility and permeability problems. Their amphiphilic characteristics allow the incorporation of lipo- and hydrophilic drugs along with the penetration across the skin and biological membranes, thermodynamic stability, promotion of drug solubilization, and sustained release [7]. All of these advantages may result in an increased bioavailability of the drugs [8]. Therefore, lyotropic liquid crystals can be beneficial in cosmetic and pharmaceutical areas.

The liquid crystalline systems are organized on the nanometer scale. The thermodynamic stability is attractive for production and scale up methods. The viscosity and texture of these systems can be varied by adjusting the ratios of the surfactant as well as the oily and aqueous phases. The versatility to control the stiffness of the systems can be useful to localize the formulation at specific sites in the body. Therefore, surfactant systems have great potential as topical nanostructured drug delivery systems [9].

Most of the surfactant-based liquid crystals reported in the literature are composed of glycerol monooleate [10,11]. Recently, we demonstrated the use of a polyoxypropylene (5) polyoxyethylene (20) cetyl alcohol (PPG-5-CETETH-20) surfactant-based system as a potential mucoadhesive agent for nasal and topical applications [12,13,14]. The oily phases including oleic acid (OA) and mineral oil play a major role in the behavior of such systems.

Polyoxyethylene (20) cetyl ether (CETETH-20) is also a surfactant that is suitable for microemulsion and liquid crystal formation in mixtures with water and different types of oily phases, such as mineral oil, oleic acid, and PEG-12-dimethicone [9]. It is possible to modulate the nanostructure, viscoelasticity, stiffness, and adhesion of such systems by only changing the ratios of the components. The adhesive properties open new avenues for developing bioadhesive formulations. Moreover, the formulations did not increase the number of leucocytes or fibroblasts in the dermis. Therefore, it can be concluded that the formulations do not cause skin irritation [9]. 

Furthermore, this study aims to determine the application of the lyotropic liquid crystalline phase behavior of CETETH-20 to obtain a potential DXM delivery system. The phase behavior of CETETH-20 in the presence of oleic acid (OA), *N*-methylpyrrolidone (P), and isopropyl myristate (IM) was investigated. Systems were characterized physically by polarized light microscopy (PLM), small-angle X-ray scattering (SAXS), rheology, texture profiles, and determination of the bioadhesive forces. In vitro release studies of the formulations were performed with a diffusion cell apparatus and the kinetics were evaluated using mathematical models. 

## 2. Materials

The surfactant CETETH-20 is commercially available as Brij 58 (Sigma-Aldrich, Steinheim, Germany). OA was acquired from Synth (Diadema, Brazil), P from Neon (São Paulo, Brazil), and IM from Vetec (Duque de Caxias, Brazil). DXM was purchased from Purifarma (São Paulo, Brazil). High-purity water was prepared with a Millipore Milli-Q plus purification system (Billerica, MA, USA).

## 3. Methodology

### 3.1. Ternary Phase Diagram Construction

**Figure 1:**
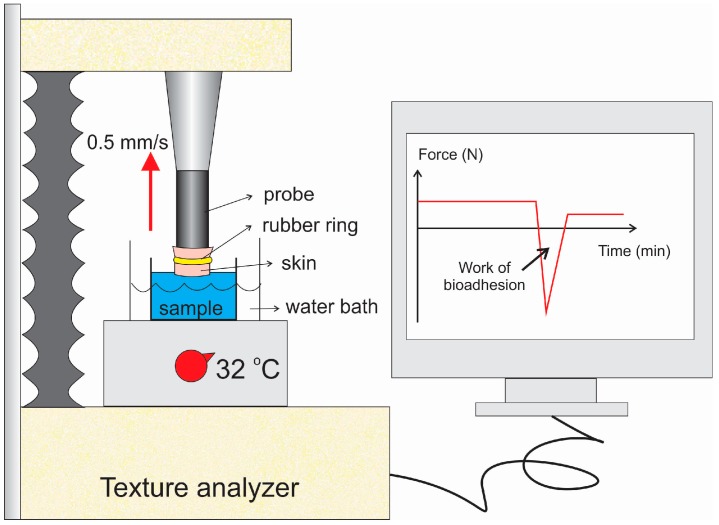
Scheme showing how the skin was adapted for the textural analyzer for the bioadhesion study.

The surfactant CETETH 20 was mixed with three different oils including OA, P, and IM in addition to water resulting in three distinct phase diagrams. A fourth diagram was constructed using the surfactant combined with P (9:1 *w*/*w*). The mixtures (surfactant and oily phase) were used in weight ratios from 1:9 to 9:1 to obtain the ternary phase diagrams. Water was slowly added using a micropipette to the mixtures (1.0 g) in a 60 °C bath to melt the surfactant. The transition from translucent dispersions (TD) to semi-solid transparent systems (SSTS) and isotropic transparent liquid systems (ITLS) or phase separations (PS) was rapid and reproducible upon the addition of 0.5 mL water. The titrations were performed at a controlled temperature of 25 ± 0.1 °C. After each phase transition, the samples were maintained at 25 ± 0.1 °C for 24 h to complete system equilibration where no phase transition was observed. The percentages of the components (*w*/*w*) after each round of water addition were calculated to obtain the points that define the boundaries between the ternary phase diagram regions. OA1, OA2, OAP1, OAP1, IM1, IM2, P1, and P2 (points, Figure 1) were chosen for further characterization tests. Their compositions are shown in Table 1 and were prepared by weighing each component, which was mixed with a glass stick in clear glass vials in a 60 °C bath to melt the surfactant. After cooling, the vials were sealed with a rubber cork.

### 3.2. Polarized Light Microscopy (PLM)

Samples were prepared by placing a drop of the formulation between a cover slip and glass slide. They were then examined under polarized light. An Optical Jenamed 2 Carl Zeiss microscope (Jena, Germany) was used to analyze the various fields of each sample at room temperature. The isotropic or anisotropic behavior of the samples was observed, and pictures were taken at a 20,000× magnification at room temperature.

### 3.3. Stability Tests

DXM was incorporated into samples at 0.1% (*w*/*v*) and they were maintained under storage conditions for six months at room temperature. Then, the formulations were centrifuged at 3000 rpm for 30 min. PLM was used to evaluate changes such as color, phase separation, pH, and mesophase identification. 

### 3.4. Rheological Analysis

The rheological sample analysis was performed at 32 °C with a controlled stress rheometer (AR2000ex, TA Instruments, New Castle, DE, USA) using parallel plate-plate geometry, 40 mm diameter steel plates, and a 0.5 mm gap. Oscillatory analyses were performed after the determination of their linear viscoelastic region at 32 °C, in which stress was directly proportional to strain, and the storage modulus remained constant. A frequency sweep analysis was performed at 0.1–100 Hz after the application of constant 5 Pa stress. Formulation samples were carefully placed on the lower plate to ensure that sample shearing was minimized, and they were allowed to equilibrate for at least 5 min before analysis.

### 3.5. Small-Angle X-ray Scattering (SAXS)

The measurements were performed at the D11 station of Synchrotron SAXS beamline at the National Laboratory of Synchrotron Light (LNLS, Campinas, Brazil). The beamline presents an asymmetrically cut and bent Si (1 1 1) monochromator (*λ* = 1.608 Å) to produce a horizontally focused beam. The temperature of the cells was 25 °C. A vertical position-sensitive X-ray detector and a multichannel analyzer were used to record the SAXS intensity, I (*q*), as a function of the scattering vector, *q*. The scattering vector, *q*, was calculated by the equation *q* = (4π/*λ*)sin(*ε*/2), where *ε* is the scattering angle. The parasitic scattering formed by slits was deducted from the total scattering intensity. SAXS curves were fitted using Sigma Plot software.

### 3.6. Texture Profile Analysis (TPA)

The measurements were performed using a TA-XTplus texture analyzer (Stable Micro Systems, Surrey, UK). Fifteen grams of each sample (three replicates) was placed in 50 mL tubes (Falcon, BD, Franklin Lakes, NJ, USA) for centrifugation to eliminate air bubbles. The tube containing the sample was fixed below the analytical probe (10 mm in diameter), which moved downward at a constant speed (0.5 mm/s) until penetrating 10 mm into the sample, and then came back to the surface. After 5 s, the movements were repeated, and finally, the probe returned to the starting position. During the test, a force-time curve was obtained from which mechanical parameters were calculated, including hardness, compressibility, adhesiveness, and cohesion. The experiments were analyzed at room temperature (25 °C).

### 3.7. Bioadhesion Studies

This study was performed using a TA-XTplus texture analyzer (Stable Micro Systems, Surrey, UK) and pig ear skin, according to previous studies of Carvalho et al. [13]. The skin model was obtained from healthy six-month-old Brazilian pigs from a local slaughterhouse. The ears were cleaned with water, and only undamaged ears were used. The skin and cartilage were removed with a scalpel. A dermatome (Nouvag TCM 300, Goldach, Switzerland) was used to obtain a 400 μm thick layer of skin that was separated from the adipose tissue. The prepared skins were stored at −20 °C. 

The experiment is schematically represented in Figure 1. The skin was maintained in physiological saline solution at room temperature (25 °C) for 30 min; then, the hair was cut and it was fixed with a rubber ring to the cylindrical probe (10 mm diameter) of the equipment. The samples were placed in 3 mm diameter flasks right below the probe, which was immersed in a 37 °C water bath. 

The test started by moving the probe down at a constant speed (1 mm/s) until the skin touched the surface of the sample and made contact for 60 s. Then, the probe moved upwards (0.5 mm/s) until the start position and contact between the surfaces was broken. A force-time curve was recorded, and the area under the force-distance curve during the withdrawing phase was calculated as the work of adhesion (WA). Samples were analyzed in triplicate at room temperature.

### 3.8. In Vitro Non-Rate Limiting Drug Release

In the drug release study, a 45 μm pore size cellulose acetate membrane was used to separate the formulation from the release medium. The test was performed using a Franz diffusion cell system (Microette Plus, Hanson Research, Chatsworth, CA, USA), where a non-rate limiting membrane of regenerated cellulose (molar mass cut-off of 12–14 kDa, previously treated with Milli-Q water at 100 °C for 5 min) was mounted on Franz cells (diffusion area of 1.77 cm^2^). The Franz cells were filled with 7 mL monophosphate buffer 0.01 M (pH 7.4) and stirred at 300 rpm. Due to the poor aqueous solubility of DXM, it was added to the receptor phase (5% *w*/*v*) of PPG-5-CETETH-20 (Procetyl AWS, Croda, Campinas, Brazil) surfactant. Three-hundred milligrams of the formulations containing 0.1% (*w*/*w*) of the drug (dose of topical formulations on the market) was applied to the surface of the membrane. The receptor phase remained under constant stirring at 37 ± 0.5 °C, and it was sampled at fixed times over 24 h. The samples (2 mL) were then filtered through a 0.45 μm cellulose acetate membrane and injected into an HPLC system. Six replicates were performed for each condition. 

### 3.9. Mechanism and Mathematical Modelling of Drug Release

The drug release profiles were analyzed by mathematical models described in the literature to elucidate the mechanisms involved in the release of DXM. The drug release curves were fitted using Hixson-Crowell, Weibull, Higuchi, Baker-Lonsdale, Korsmeyer-Peppas, and Hopfenberg models, and the adjusted coefficient of determination (Equation (1)) was calculated, which was used to choose between the models.

(1) $$R_{\mathrm{adjusted}}^{2}=1-\frac{\left(n-1\right)}{\left(n-p\right)}(1-R^{2})$$

where *n* is the number of dissolution data points, and *p* is the number of parameters in the model. The measured values obtained in the drug release assay were adjusted to these mathematical models.

### 3.10. Analytical Method

In this study, DXM was analyzed by HPLC with a UV detector (Varian Pro Star 330) set at 239 nm as it was previously detected by a UV sweep. The test conditions included a C18-reversed phase column (250 × 4.6 mm i.d., 5 μm pore size) (Phenomenex Inc., Torrance, CA, USA), a mobile phase of methanol, acetonitrile, and water (35:35:30 *v*/*v*), a flow rate of 0.8 mL/min, and an injection volume of 50 μL. A calibration curve was constructed using standard solutions of DXM in methanol at concentrations ranging from 0.1 to 30 μg/mL.

## 4. Results

### 4.1. Phase Behavior and Physicochemical Characterisation

**Figure 2:**
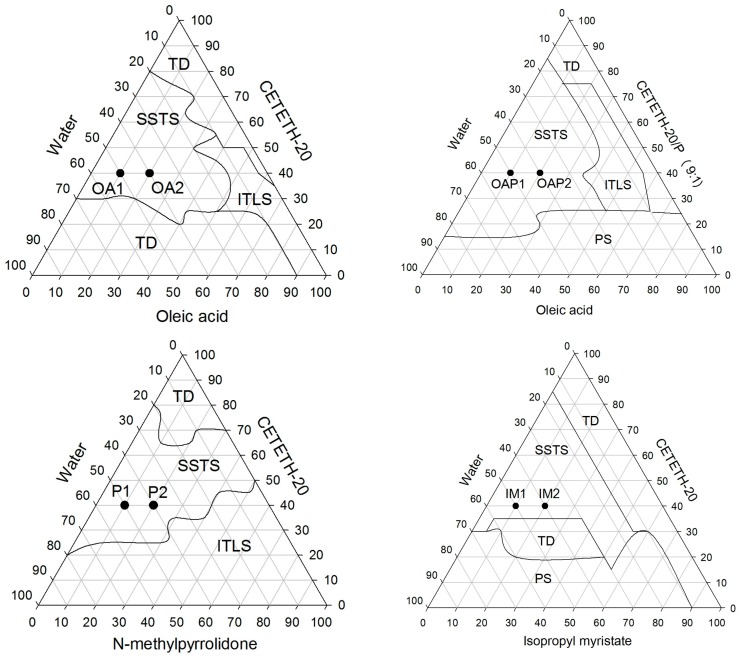
Ternary phase diagrams of CETETH 20 combined with oleic acid (OA), *N*-methylpyrrolidone (P), and isopropyl myristate (IM) as oily phases. OA1, OA2, IM1, IM2, OAP1, OAP2, P1, and P2 were the points chosen for characterization. (TD) translucent dispersion, (SSTS) semi-solid transparent systems, (ITLS) isotropic transparent liquid system, and (PS) phase separation.

A phase diagram is an excellent tool to provide an overview of where phase transitions may occur. The ternary phase diagrams constructed for the oily phases including OA, P, and IM are presented in Figure 2. The systems, termed semi-solid transparent systems (SSTS), were visually classified as potential liquid crystals when they were semi-solid, exhibited transparency, were spontaneously formed, and had thermodynamic stability. The SSTS occurred over a wide range of surfactant concentrations (from 20 to 90%) and may be composed of straight and reverse structures since it was possible to obtain SSTS with oily phases of approximately 1–50%.

Physico-chemical studies were performed for formulations (1) and (2), as indicated in the ternary phase diagrams in Figure 2, and their compositions are listed in Table 1. The concentration of the surfactant (CETETH-20) was fixed at 40% (*w*/*w*), whereas the other concentrations varied. Results of the stability tests showed that the formulations kept their homogeneity, color, mesophase, and pH (3.51–4.91).

**Figure 3:**
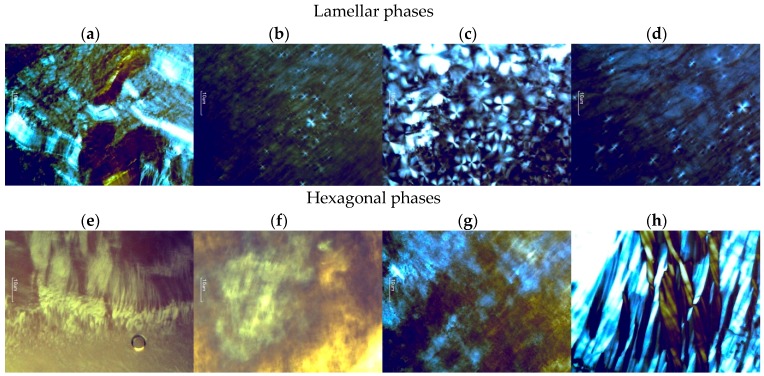
Photomicrographs obtained using polarized light microscopy (PLM) that shows the streaks of the hexagonal phases (IM1, IM2, P1, and P2) and Maltese crosses of the lamellar phases (OA1, OA2, OAP1, and OAP2). The sample compositions are described in Table 1; (**a**) OA1; (**b**) OA2; (**c**) OAP1; (**d**) OAP2; (**e**) IM1; (**f**) IM2; (**g**) P1; (**h**) P2.

Lyotropic liquid crystals that are anisotropic and rotate the polarization plane result in various patterns of colors and texture. Hexagonal structures lead to fan-like images and lamellar phases have “strip-like” structures [15] as can be observed in Figure 3. In this way, samples P and IM are potential hexagonal phases and OA and OAP can be lamellar phases.

**Figure 4:**
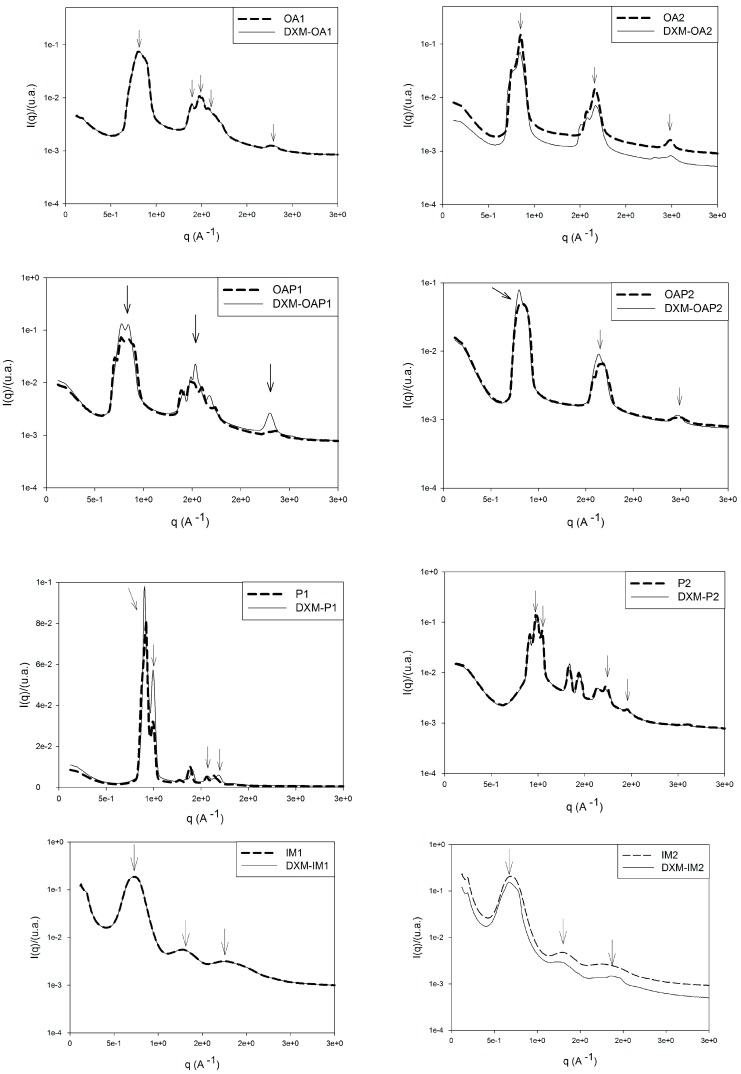
Small-angle X-ray scattering (SAXS) patterns of the formulations. The arrows indicate the considered peaks for the mesophase classifications.

The formation of various mesophases with different textures can be observed. Macroscopic and microscopic observations cannot be the only assessment criterion for lyotropic liquid crystals but should be verified using additional methods [15]. To achieve this, the phase behavior of the liquid crystals was also evaluated by SAXS. The curves obtained from the plot of intensity, I, versus the scattering vector module, *q* (Å^−1^), are shown in Figure 4. Structures formed by lyotropic liquid crystals can be interpreted from the peak positions on the scattering vector axis. 

**Figure 5:**
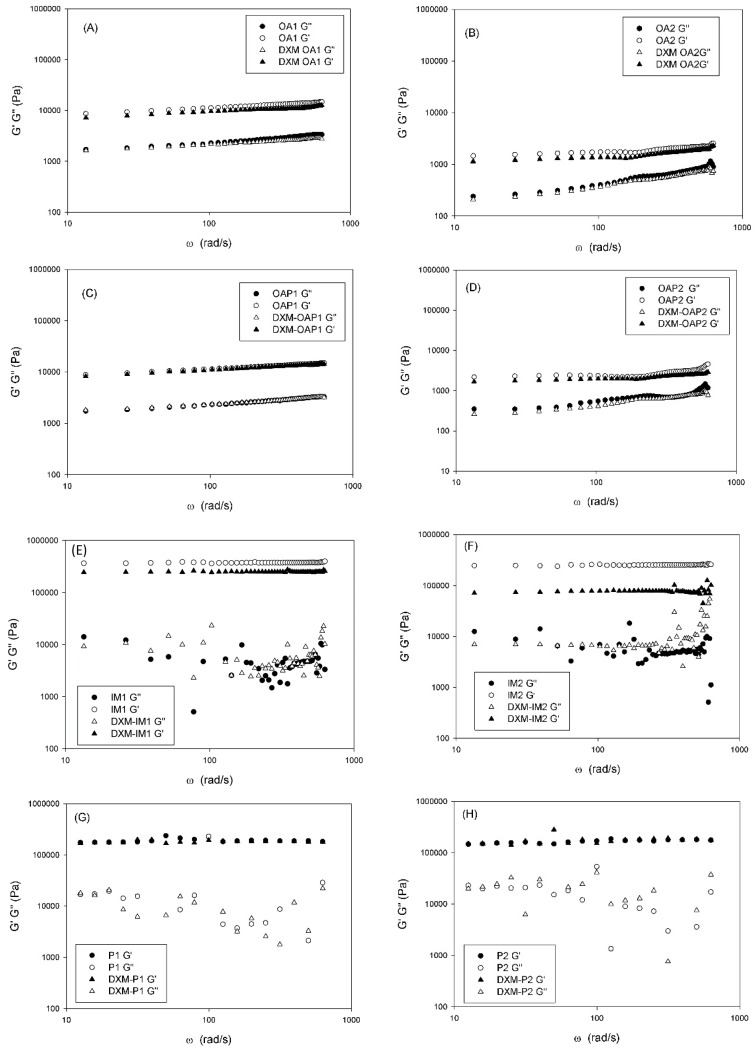
The frequency sweep profile of the storage (G′) and loss moduli (G′′) of samples at 25 °C. DXM loaded formulations. The sample compositions are described in Table 1.

As seen in Table 2, the ratio between the first and second peaks of formulations OA2, OAP1, and OAP2 is 2:1, which is characteristic of lamellar phases [16]. These results confirm the Malta crosses visualized by PLM for these lamellar phases. For hexagonal liquid crystals, the relative peak positions (compared to the most intense peak) should adhere to 1:√3:2:√7 [16] as observed in the scattering sample profiles of IM1, IM2, P1, and P2 (Figure 4). The texture of the hexagonal phase that was visualized by PLM was confirmed for the P and IM formulations; however, Maltese crosses for OA1 were also observed. In addition, the oscillatory rheological profile of this sample (Figure 5) also confirms the phase behavior. Since the SAXS curve of OA1 presented an overlap of the second peaks, a mesophase classification was considered for the PLM and rheological measurements of this sample.

The correlation distance between the scattering objects was calculated as d = 2π/*q_max_*, where *q_max_* is the *q* value at the intensity peak I (*q*), and d is the lattice parameter. For lamellar phases, *d* is the spacing between two adjacent layers. For hexagonal phases, *d* is the distance between adjacent rows of cylinders in the hexagonal structure [17]. Table 2 lists the calculated *d* values for the formulations. The results indicate that the distances, *d*, between the scattering objects are in a nanoscale range since 1 Å = 0.1 nm. Therefore, the liquid-crystalline systems present a nanostructured organization. 

Data are shown for DXM loaded formulations as well, and demonstrate that the drug did not alter the structure of samples, except for loaded DXM-IM2, in which the correlation peaks of the hexagonal phase was loosened (*d*_1_/*d*_2_ = 1.94: Table 2). This correlation value was not reported in the literature to allow for a mesophase classification.

The hexagonal phase is formed by cylindrical micelles. The cross section of the micelles can be round or oval. The lamellar phase is formed by the bilayers of two planes with non-penetrating or mutually penetrating hydrocarbon chains. The length of the planes is limited by the size of the container [15].

In addition to transparency, rheology was also utilized for the liquid crystal investigation. Visually, the viscosity of the hexagonal phases may be so high that the liquid does not flow. A decrease in viscosity compared with the hexagonal phases is a characteristic feature of the presence of the lamellar phase. The viscosity drop is caused by the layer structure of the phase, which results in easy slip planes [15]. The oscillatory rheological data confirm these properties. Figure 5 shows the rheograms of the storage (G′) and loss moduli (G′′) as a function of the oscillatory frequency. 

The storage modulus represents the solid-like component of a viscoelastic material. The loss represents the liquid-like element [13,18]. The frequency sweep analyses suggest that OA and OAP samples are more elastic and less viscous at the selected frequency because G′ > G′′. Furthermore, both moduli are frequency dependent, since a small slope can be observed on the rheological curves. Independent of the frequency, the G′ values were approximately 100× higher for the P and IM samples than for the OA and OAP samples. This indicates a highly structured character. 

Although OA1 samples have been classified as hexagonal phases by SAXS, the Maltese crosses characteristic of lamellar phases was visualized by PLM, and the rheological profile follows the same pattern of the lamellar phases of OA2, OAP1, and OAP2 suggesting that OA1 is a lamellar liquid crystal.

Oscillatory rheology performed in the linear viscoelastic region can give information about the microstructure of the system at rest. In low-frequency ranges, an entangled network can disentangle according to time, while a network of secondary bonds is fixed independent of the time. For dispersions of high molecular weight, G′ > G′′ represents a network consisting of secondary bonds and G′ ≤ G′′ for physically entangled systems. The curve of viscoelastic moduli versus frequency of an entangled system shows a slope, while for a network of secondary bonds constant values of moduli are observed all over the frequency range [18]. According to the rheological curves shown in Figure 5, OA and OAP samples present a slight slope, while P and IM samples present a frequency independent value of G′ and complete loss of the viscous component. In addition, it can be seen that the loss modulus G′′ of the lamellar phases OA and OAP present a linear trend, while this trend was lost for the hexagonal phases P and IM. The high values of G′ and the plateau region presented by P and IM samples are related to the formation of an elastic structural network due to the interactions between the liquid-crystalline domains, which is stronger for hexagonal than lamellar phases. This phenomenon is traduced macroscopically by the flowability of the lamellar phases and the extremely high stiffness of the hexagonal phases.

In the lamellar phases, OA1 and OAP1 exhibited higher G′ values than the OA2 and OAP2 systems. The main difference between group 1 and 2 is the balance between the proportions of aqueous and oily phases. Lamellar phases with higher water content (group 1: OA1 and OAP1) resulted in stronger interactions between the bilayers than lamellar phases with the same amount of surfactant, but a lower proportion of water (group 2: OA2 and OAP2). It can be suggested that the increase in the oily phase results in weaker interactions between the bilayers and the H-bonds between surfactant and water and may improve the strength of the bilayer network.

The difference between the proportions of the components of the formulations did not alter the elastic behavior of the hexagonal samples and the G′ values were of the same magnitude. However, for loaded IM2, the drug may have interfered in the network, since there was a pronounced decrease in G′. This points to structural changes and is consistent with the SAXS results since the DXM-loaded IM2 lost its hexagonal SAXS pattern. However, the magnitude of G′ is still higher than in the lamellar phases. Therefore, both hexagonal and lamellar phases were found to be more elastic than viscous in the range of frequency considered, and the storage modulus was higher than the loss modulus. Moreover, the elastic properties of the hexagonal phase were higher than that of the lamellar phase.

The hexagonal phase presented the strongest network and the most stable internal structure as it presented higher G′ values. This corresponds to high-density connection points of the cylindrical micelles and strong interactions developed within and among the monodomains [19]. 

The PLM, SAXS, and rheological measurements show that DXM did not alter the structure of the liquid crystals in general. Since it is a lipophilic drug, it may be stabilized and protected in the oily phases.

### 4.2. Textural Analysis and Skin Bioadhesion Strength

**Figure 6:**
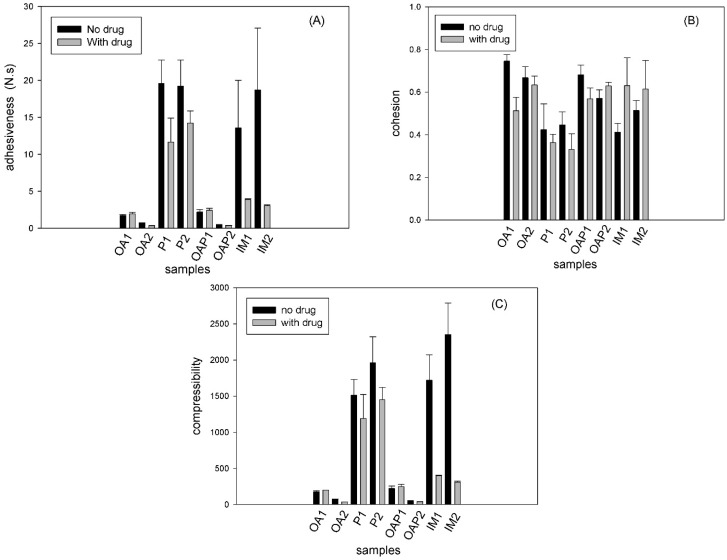
Textural parameters obtained using the textural profile analysis (TPA) test. OA and OAP: lamellar phases, P and IM: hexagonal phases. The compositions are described in Table 1.

The mechanical properties of the formulations, such as hardness, compressive resistance, cohesion, and adhesion were measured using a texture analyzer. The parameters obtained were calculated from force time curves. The results are shown in Figure 6.

Compressibility is the total force area obtained during the first compression cycle in the TPA test (Stable Micro Systems). The compressibility results (Figure 6C) exhibited the same trend as the adhesion parameter (Figure 6A). Adhesion was calculated from the negative area of the force-time curve, which was generated during the first compression of the TPA test. Adhesion is the work required to overcome the attractive forces between the probe and sample.

Figure 6A,C show that the hexagonal phases (P and IM samples) were found to be harder and more adhesive than the lamellar phases (OA and OAP samples). These data correlate with the rheological analysis in Figure 5, where the elastic modulus of the hexagonal phases was higher than in the lamellar phases.

The drug did not significantly interfere in the textural behavior of all OA, OAP, and P samples, which correlates with the SAXS and rheology results that show that the network integrity is maintained for loaded samples. However, the mechanical parameters decreased for the loaded IM1 and IM2. Moreover, this result correlates with the SAXS result, where the lattice parameters calculated for the loaded IM2 show a loss of hexagonal pattern (Table 2). Furthermore, the rheology result of the loaded IM1 and IM2 shows a decrease in the elastic modulus in Figure 5E,F. 

Cohesion is the rate at which the sample disintegrates under mechanical action. Cohesion occurs due to the ratio between the positive area of the second compression and the area measured in the first compression. The results in Figure 6B show that there was a loss of sample structure and this behavior is an indication of shear thinning, which is desirable for topical pharmaceutical dispersions because the decrease in stiffness (shear thinning) facilitates topical application.

**Figure 7:**
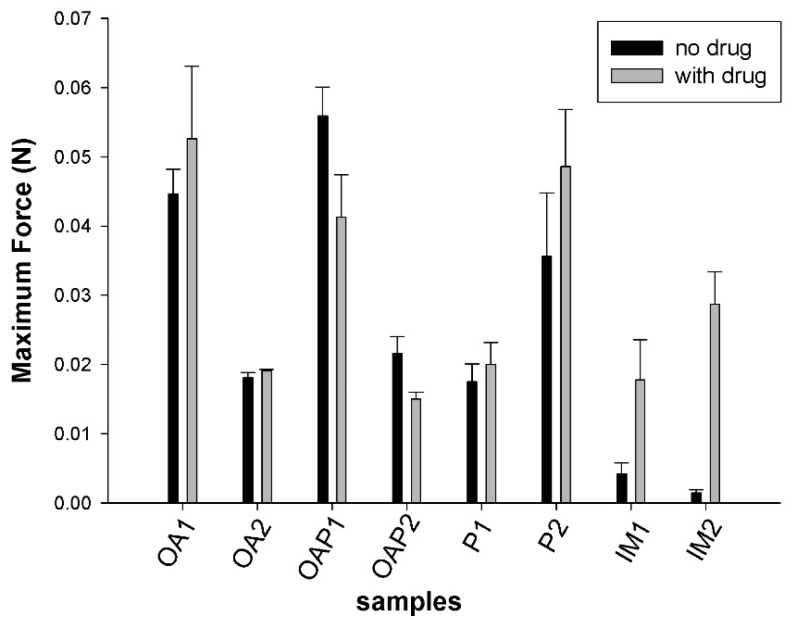
Results of the ex vivo skin bioadhesion test. Data were collected at 25 ± 0.5 °C. The values represent the mean ± the standard deviation (S.D.) of three replicates. OA and OAP: lamellar phases, P and IM: hexagonal phases. The composition is described in Table 1.

The measurements of the skin bioadhesion strength are shown in Figure 7. Although the mean work of the bioadhesion values of DXM loaded formulations was higher than in unloaded samples the differences were not statistically significant. 

Unexpectedly, the skin bioadhesion behavior did not follow the rheological and textural behavior. There was no relationship between the mesophase and force of bioadhesion. It is suggested that the bioadhesive force is more dependent on the composition of the systems than the mesophase network. Samples containing OA in the oily phase (OA and OAP) were less adhesive when a lower amount of oil was present (OA1 = OAP1 > OA2 = OAP2). In this case, it can be suggested that the oil conferred a lubricant property that decreases the interaction with the skin. Samples composed of IM presented low values of bioadhesion that can be a result of their position in the ternary phase diagram (Figure 2), which is localized close to the phase transition line. The conditions in which the test was performed offer additional external parameters, such as the composition of the skin and elevation of temperatures to 32 °C, which may interfere with the stability of these samples and can result in phase transitions. Moreover, weakness to preserve the stability of the samples was revealed in the TPA test since the cohesion values were the lowest in the IM samples. Finally, for formulations where the oily phase was substituted by P, a dipolar aqueous and permeation enhancer agent [20], the bioadhesion was higher for formulations containing more of the component. This test was critical to show how in vivo conditions may influence the performance of the formulation. It can be suggested that oils may decrease bioadhesion while hydrophilic permeation enhancer agents increase this property. It was also shown that even a stiff formulation could lose its bioadhesion if it is not stable enough. 

### 4.3. Drug Release Assay and Mathematical Modelling 

**Figure 8:**
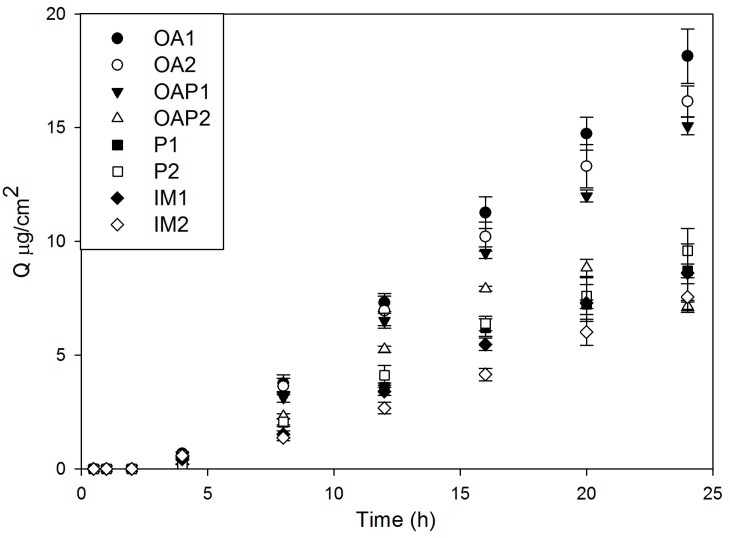
Release profile of DXM-loaded liquid crystal formulations (0.1% *w*/*w*) described in Table 1.

The DXM released was expressed as μg/cm^2^, which was plotted against time in Figure 8. As demonstrated, the curve is convex to the time axis at the early stage and then becomes linear. This early stage is a non-steady state condition. Later, the rate of diffusion is constant, the curve is essentially linear, and the systems are at a steady state [21]. 

The drug release profiles exhibited a lag time, which is a measure of the time it takes for the permeant concentration gradient to become stabilized across the membrane. It can be obtained by the intercept of the steady-state line [21,22]. The results of the calculated lag time formulations are in Table 3. It takes approximately 3 h for all formulations to reach the steady state condition, which is a good indication of prolonged drug release. 

Kinetic evaluations were performed by plotting the results as the mean cumulative amount of the drug against time. Table 3 shows the adjusted coefficient of determination (R^2^) resulting from the fitted curves using the drug release mathematical models described by Hixson-Crowell, Weibull, Higuchi, Baker-Lonsdale, Korsmeyer-Peppas, and Hopfenberg [23]. The higher the R^2^ is, the better the model can be applied to interpret the drug release profile. Table 3 shows that the mathematical models that best fit the drug release curves are the Korsmeyer-Peppas and Weibull, as they resulted in R^2^ > 0.99.

The Korsmeyer-Peppas model is represented by the Equation (2):

(2) $$\frac{M_{t}}{M_{\infty }}=K\;t^{n}$$

where *M_t_*/*M_∞_* is a fraction of the drug released over time, *t* (h). The release exponent is represented by *n*, and *K* is the release rate constant. The *n* exponent is characteristic of the release mechanism. Normal Fickian diffusion is characterized by *n* = 0.5 and case II diffusion by *n* = 1.0. A value of *n* between 0.5 and 1.0 indicates a mixture of Fickian and case II diffusion, which is usually called non-Fickian, or anomalous diffusion. The values of all liquid crystalline samples were *n* > 1, which means both erosion and diffusion may control the drug release [23].

The Weibull equation expresses the accumulated fraction of the drug, m, in the solution at time, t, by:

(3) $$m=1-\exp\left[\frac{-{\left(t-\mathrm{Ti}\right)}^{b}}{a}\right]$$

In this equation, the scale parameter, a, defines the time scale of the process. The location parameter, Ti, represents the time lag before the onset of the dissolution or release process and in most cases will be zero. The shape parameter, b, characterizes the curve as exponential, sigmoid, S-shaped (with upward curvature followed by a turning point), or parabolic (with a higher initial slope and after that consistent with the exponential curve) [23]. Table 3 shows the b values derived from the fitting of Equation (3) to the 60% drug release curve data. It can be observed that all samples presented b > 1, which is because of the sigmoid shape of the Weibull function and indicates that a complex mechanism governs the release process [24]. The release rate initially increases nonlinearly up to the inflection point and after that increases linearly.

The analyses of both the Korsmeyer-Peppas and Weibull models indicate that the liquid crystals do not follow simple diffusion or erosion, and a combination of mechanisms is involved. 

The liquid crystalline matrix is a two-phase system, with the oily and aqueous phases stabilized by the surfactant, forming regions with different dielectric constants, which interfere with drug diffusion and erosion of the matrix. Since PLM, SAXS, and rheology results showed the drug did not alter the structure of the systems in general, it was suggested that lipophilic DXM is solubilized and protected in the oily phase of the systems. The drug has to overcome the hydrophilic domains of liquid crystalline structures to be released, which may explain the lag time of drug release curves. The lamellar phases (OA and OAP samples) allowed a higher amount of drug release than the hexagonal phases (P and IM samples) because of their lower network crosslink. The mobility of the molecules may be lower in the hexagonal phases than the lamellar phases. On the contrary, both the lamellar and hexagonal phases prolonged the drug release.

These findings are correlated with other results from our group that determined the drug kinetics in liquid crystalline matrices composed of PPG-5-CETETH-20, OA, and water [14,25]. Carvalho et al. (2009) determined that the zidovudine kinetics in the lamellar phase decrease the drug release considerably when compared with a microemulsion made of the same components [25]. The lamellar and cubic phases of those components also controlled and extended the delivery of fluconazole [14]. The lamellar phases promoted the retention of fluconazole in the skin, while the cubic phases promoted transdermal transportation through the skin. It has been reported that the concentration of antifungal drugs attained in the skin is an important factor in the treatment of dermatomycosis and that the presence of the therapeutically active form in the skin is closely related to the efficacy of the drug [14]. This study showed that it is possible to promote drug permeation or skin retention by changing only the composition of the components in the formulations. It is important to point out that both DXM and fluconazole are lipophilic and zidovudine is hydrophilic, showing the versatility of lyotropic liquid crystals in controlling the release of drugs with different solubilities.

These findings have led the way to consider CETETH-20-based lyotropic liquid crystals for skin delivery. Typically, transdermal systems do not have a lag time, since the drug may not be released over a significant time during use, and keeping track of the blood levels may be almost impossible [22]. According to our results, the formulations presented an average of 3 h of lag time, which may discourage the transdermal application of these systems. On the contrary, it presents great potential for oral, mucosal, and wound healing applications and can be considered as a scaffold for osteogenic differentiation. The stiffness of liquid crystals may improve the mucoadhesion and fixation of formulations at the site of action. In this case, the prolonged release of the drug is desired for depot systems.

CETETH-20 systems can be considered promising drug delivery systems for DXM incorporation, because they can be developed in a gel-like form with different viscosities by varying the amount of each component. Moreover, the various ways presented individual control release profiles. This property is advantageous for designing new preparations for various routes of administration, such as oral and mucosal, since they also present bioadhesive properties.

Therefore, lyotropic liquid crystals composed of CETETH-20, OA, P, IM, and water are thermodynamically stable and form spontaneously. Such properties represent great appeal for scaling up since their low-cost production requires no specific equipment for preparation. In this way, this work represents a starting point for future development of formulations for controlled delivery systems for DXM.

## 5. Conclusions

Systems formed with CETETH-20 based compositions may form into liquid crystalline structures, as shown by PLM and SAXS measurements. The rheological analysis revealed that the hexagonal phases present a higher storage modulus than the lamellar phases. These systems promoted bioadhesion and controlled DXM release in an in vitro study. These novel drug delivery systems are suitable for pharmaceutical applications since they require simple methods for preparation. CETETH-20-based systems may be a promising approach for DXM delivery considering the simplicity of the technology and the controlled DXM release profile.

## Figures and Tables

**Table 1:** Composition of the liquid crystal formulations (%, *w*/*w*).

	OA1	OA2	OAP1	OAP2	IM1	IM2	P1	P2
CETETH-20	40	40	40	40	40	40	40	40
Oleic acid	10	20	9	19	-	-	-	-
*N*-methylpyrrolidone	-	-	1	1	-	-	10	20
Isopropyl myristate	-	-	-	-	10	20	-	-
Water	50	40	50	40	50	40	50	40

**Table 2:** Peak positions (*q*) of the small-angle X-ray scattering (SAXS) curves, their respective correlation distances (*d*), and assignments for phase classification (*d*_1_/*d*_2_, *d*_1_/*d*_3_). DXM stands for dexamethasone loaded formulations. The sample compositions are described in Table 1.

Samples	*q*_1_ (Å^−1^)	*q*_2_ (Å^−1^)	*q*_3_ (Å^−1^)	*d* (Å)	*d*_1_/*d*_2_	*d*_1_/*d*_3_	Classification
OA1	0.80	1.38	1.43	7.85	1.73	1.79	*
DXM-OA1	0.80	1.38	1.43	7.85	1.73	1.79	*
OA2	0.84	1.65	2.47	7.48	1.96	2.94	Lamellar
DXM-OA2	0.83	1.66	2.48	7.57	2.00	3.00	Lamellar
OAP1	0.76	1.52	2.29	8.26	2.00	3.00	Lamellar
DXM-OAP1	0.77	1.53	2.29	8.15	2.00	2.97	Lamellar
OAP2	0.80	1.65	2.49	7.85	2.00	3.10	Lamellar
DXM-OAP2	0.78	1.63	2.41	8.00	2.00	3.00	Lamellar
P1	0.90	0.99	1.55	6.98	1.10	1.72	Hexagonal
DXM-P1	0.89	0.99	1.54	7.05	1.10	1.73	Hexagonal
P2	0.99	1.04	1.72	6.34	1.00	1.73	Hexagonal
DXM-P2	0.99	1.04	1.72	6.34	1.00	1.73	Hexagonal
IM1	0.72	1.25	1.90	8.72	1.73	2.63	Hexagonal
DXM-IM1	0.72	1.25	1.90	8.72	1.73	2.63	Hexagonal
IM2	0.72	1.28	1.75	8.72	1.78	2.43	Hexagonal
DXM-IM2	0.66	1.28	1.87	9.52	1.94	2.83	**

(*) overlapped peaks, classification not possible. (**) values of correlation peaks not found in literature for mesophase’s classification.

**Table 3:** Adjusted coefficient of determination (R^2^) obtained by the kinetic evaluation of drug release plots and calculation of lag time. (n) The Korsmeyer-Peppas exponent of the release mechanism; (b) shape parameter of Weibull model.

Kinetic Model	Parameters	OA1	OA2	OAP1	OAP2	P1	P2	IM1	IM2
	Lag time (h)	2.56	3.21	3.33	3.10	3.24	3.37	3.68	3.66
Baker-Lonsdale	R^2^	0.77	0.00	0.77	0.78	0.77	0.77	0.76	0.75
First order	R^2^	0.96	0.97	0.96	0.91	0.96	0.96	0.96	0.95
Higuchi	R^2^	0.77	0.78	0.78	0.79	0.77	0.77	0.76	0.75
Hixson-Crowell	R^2^	0.96	0.97	0.97	0.90	0.96	0.96	0.96	0.96
Korsmeyer-Peppas	R^2^	0.99	1.00	0.99	0.99	0.99	0.99	1.00	0.99
	n	1.67	1.34	1.36	1.74	1.82	1.74	1.81	1.58
Weibull	R^2^	1.00	1.00	1.00	0.99	0.99	1.00	1.00	0.99
	b	1.37	1.20	1.17	5.6	1.50	1.17	2.22	1.73

## Abbreviations

CETETH-20	polyoxyethylene (20) cetyl alcohol
OA1 and OA2	samples containing CETETH-20/oleic acid/water at 40:10:50 and 40:20:40
OAP1 and OAP2	samples containing CETETH-20/oleic acid/*N*-methylpyrrolidone/water at 40:9:1:50 and 40:19:1:40
IM1 and IM2	samples containing CETETH-20/isopropyl myristate/water at 40:10:50 and 40:20:40
P1 and P2	samples containing CETETH-20/*N*-methylpyrrolidone/water at 40:10:50 and 40:20:40
